# The role of Hfq in regulation of lipA expression in Pseudomonas *protegens* Pf-5

**DOI:** 10.1038/s41598-017-10808-x

**Published:** 2017-09-04

**Authors:** Wu Liu, Menggang Li, Jinyong Yan, Yunjun Yan

**Affiliations:** Key Laboratory of Molecular Biophysics, Ministry of Education, College of Life Science and Technology, Huazhong University of Science and Technology, Wuhan, 430074 China

## Abstract

*Pseudomonas* lipase is a well-studied lipase. However, few studies have been conducted to examine the mechanisms underlying the regulation of the lipase expression. Hfq is a global regulatory protein that, among others, controls the expression of multiple genes, regulate bacterial peristalsis, and participates in the regulation of quorum-sensing (QS) system. In this study, the effects of Hfq on lipase expression were investigated by knocking out the *hfq* and *rsmY* genes or overexpressing of *hfq* and *rsmY* genes. We found that Hfq regulates the expression of *lipA* at both transcriptional and translational levels. The translational level was the main regulatory level of *lipA*. Hfq also regulates the expression and stability of *rsmY*. Additionally, using *hfq/rsmY* double gene knock-out, we showed that Hfq can directly bind to the *rsmY* to regulate *lipA* activity. In conclusion, our results indicate that Hfq regulates the expression of *rsmY* mainly at the translational level to influence the expression of *lipA* in *Pseudomonas protegens* Pf-5.

## Introduction

Lipase, which is present in a variety of animals, plants, and microorganisms, is an important industrial enzyme^1, 2^. However commercial lipase is derived mainly from microorganisms. Currently, lipase is used in the food, beverage, oil, detergent, cosmetics, paper, pollution control, and bioenergy industries. However, conventional culture and optimization of fermentation conditions have not been able to overcome problems related to inadequate production of bacterial lipase. Therefore, it is necessary to elucidate the molecular mechanisms underlying the regulation of expression of genes involved in lipase production in order to resolve these issues.

Previous studies have shown that the expression of bacterial lipase is regulated by a two-component regulatory system^3, 4^. For instance, in *Pseudomonas alcaligenes*, LipQ*-*LipR *(lipQ/R)* directly regulates lipase expression. In *Pseudomonas aeruginosa*, CbrA-CbrB *(cbrA/B)* is the two-component regulator of lipase expression. Moreover, *las, rhl*, and other two-component regulatory systems regulate the expression of the lipase quorum-sensing (QS) system. Our previous study showed that in *Pseudomonas protegens* Pf-5, two-component regulatory system GacS*-*GacA mediates *lipA* expression via *rsmE* rather than *rsmA* ^5^. However, it remains unclear whether other lipase-regulatory genes exist in *P. protegens* Pf-5.

Hfq was first found in *Escherichia coli* as an RNA-binding protein capable of affecting a series of phenotypes of bacteria, such as growth, virulence-factor expression, and resistance^6^. Further studies showed that the small regulatory RNA (sRNA) *rsmY* is directly bound to *hfq* in *P. aeruginosa* PAO1 to maintain the stability of *rsmY*, thereby enhancing its regulation of the expression of a series of genes^7, 8^. In other *Pseudomonas sp*., *hfq* also binds to the sRNA PhrS, to stimulate the expression of resistance genes. It has also been established that *hfq* binds to the 5′-noncoding region of *pltR, pltT*, and other genes to alter their expressions^9^. These findings identify *hfq* as a global regulatory factor capable of affecting various physiological processes essential to bacterial survival.

However, in *P. protegens* Pf-5, the function of *hfq* has not been studied, and it remains unclear whether it regulates lipase gene expression. In the present study, evidence is provided showing that *hfq* knockout significantly reduces lipase production, and that *hfq* directly interacts with *rsmY* to control the expression of lipase in *P. protegens* Pf-5.

## Results

### Hfq regulates the expression of lipA at the translational level

**Figure 1:**
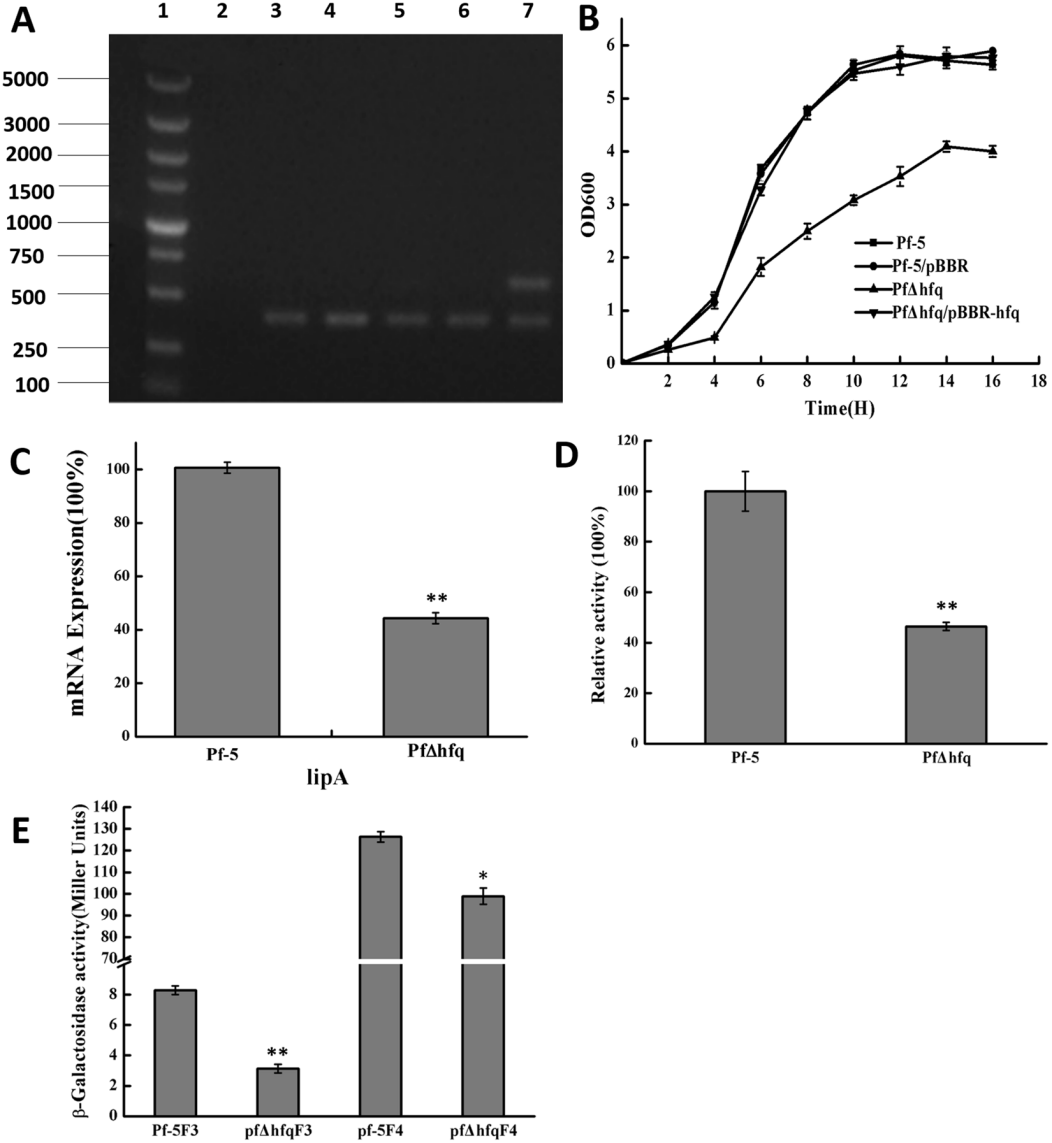
Effects of *hfq* mutation on *lipA* expression. (**A**) PCR confirmed *hqf* knockout. Lane 1: DNA markers. Lane 2: negative control (−). Lane 3, 4, 5, 6: mutant strains (PfΔhfq). Lane 7: Recombinant failed strains (**B**) Growth curve of *Pseudomonas sp*. Overnight bacterial cultures were performed in 50 mL LB. The initial OD value was adjusted to ~0.1. Bacterial cultures were shaken at 200 rpm at 28 °C, and OD value were determined once every 2 h. (**C**) qRT-PCR of relative *lipA* expression in *P. protegens* Pf-5 wild-type and the *hfq* mutant, and lipA mRNA levels were measured when bacterial growth reached the stationary phase. (**D**) Relative activity of whole-cell lipase in *P. protegens* Pf-5 wild-type and the *hfq* mutant. Whole-cell lipase activity was measured following bacterial culture in 50 mL LB to the stationary phase. (**E**) β-galactosidase activity in *P. protegens* Pf-5 wild-type and the *hfq* mutant. Bacteria were incubated in 50 mL LB to the stationary phase, and the enzyme activity of β-galactosidase was determined. Experiments were completed in triplicate. *P < 0.05, **P < 0.01 compared with the control group.

In P. protegens Pf-5, there is no classical QS regulatory system. In order to further understand the relationship between Hfq and lipA, we conducted hfq gene knockout (Fig. 1A). The effect of hfq on lipase expression was analyzed by measuring the activity of β-galactosidase and whole cell lipase. Figure 1B shows that growth of Pf-5 was decreased by the knockout of hfq (Fig. 1B). Furthermore, after the hfq knockdown, the relative activity of lipA and the mRNA expression of lipA were significantly decreased (Fig. 1C and D).

**Figure 2:**
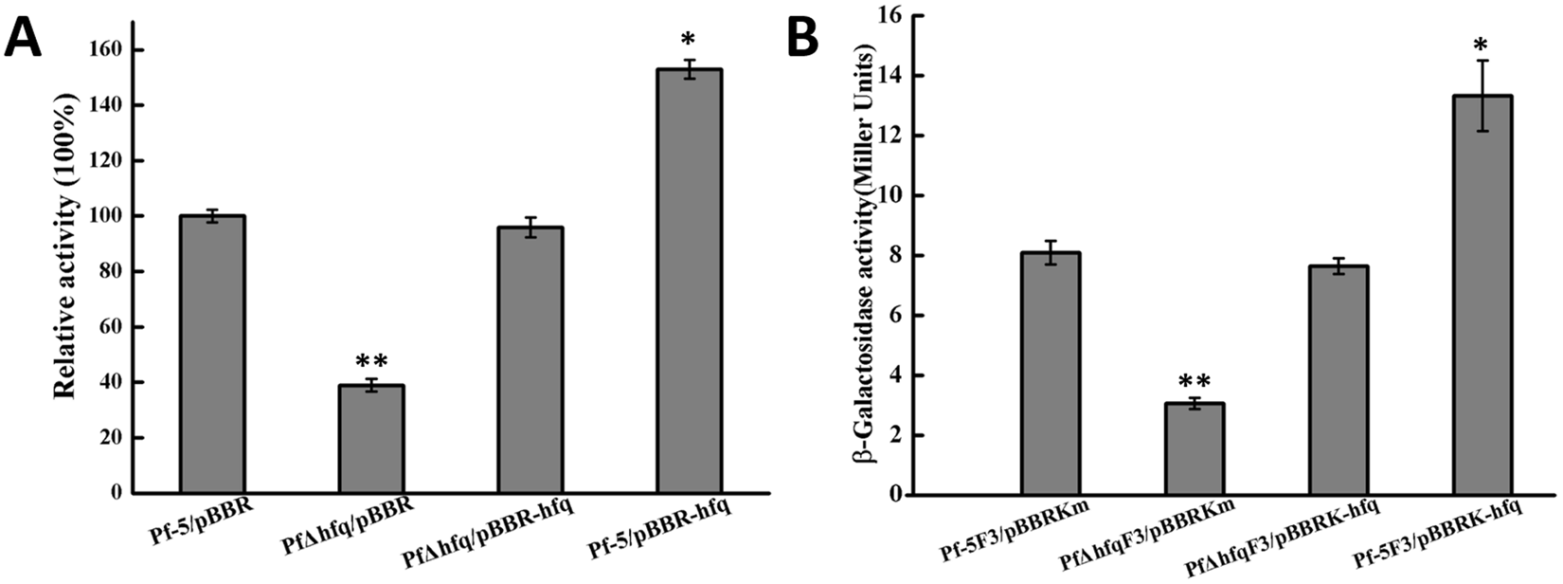
Effects of *hfq* overexpression on *lipA* expression. (**A**) Relative activity of whole-cell lipase in *P. protegens* Pf-5 wild-type and the *hfq* mutant. Whole-cell lipase activity was measured after bacterial culture in 50 mL LB growth reached the stationary phase. (**B**) Influence of *hfq* overexpression on the expression of the chromosome-borne *lipA*′*-*′*lacZ* construct in different strains. Bacteria were cultured in 50 mL LB to the stationary phase, and β-galactosidase activity was determined. Pf-5F3/pBBRKm: *P. protegens* Pf-5 wild-type with pBBRKm; Pf-5F3/pBBRK-hfq: *P. protegens* Pf-5 wild-type overexpressing *hfq*; PfΔhfqF3/pBBRK: hfq mutant with pBBRKm; and PfΔhfqF3/pBBRK-hfq: PfΔhfq complementary *hfq* mutant. Experiments were completed in triplicate. *P < 0.05, **P < 0.01 compared with the control group.

In the wild-type *P. protegens* Pf-5, when *hfq* was reconstituted with *hfq*, relative lipase activity was restored in the *hfq* mutant, indicating that *hfq* knockout affected *lipA* expression at the promoter level and hence lipase activity. Moreover, measurement of lipase and β-galactosidase activities following *hfq* overexpression in *hfq* complementation-mutant strains revealed restoration of the activities of β-galactosidase and lipase to wild-type levels (Fig. 2A and B). These results suggest *hfq* involvement in regulating *lipA* expression and lipase activity through its influence on *lipA* at the transcription and translation levels.

### Influence of Hfq on rsmY expression

**Figure 3:**
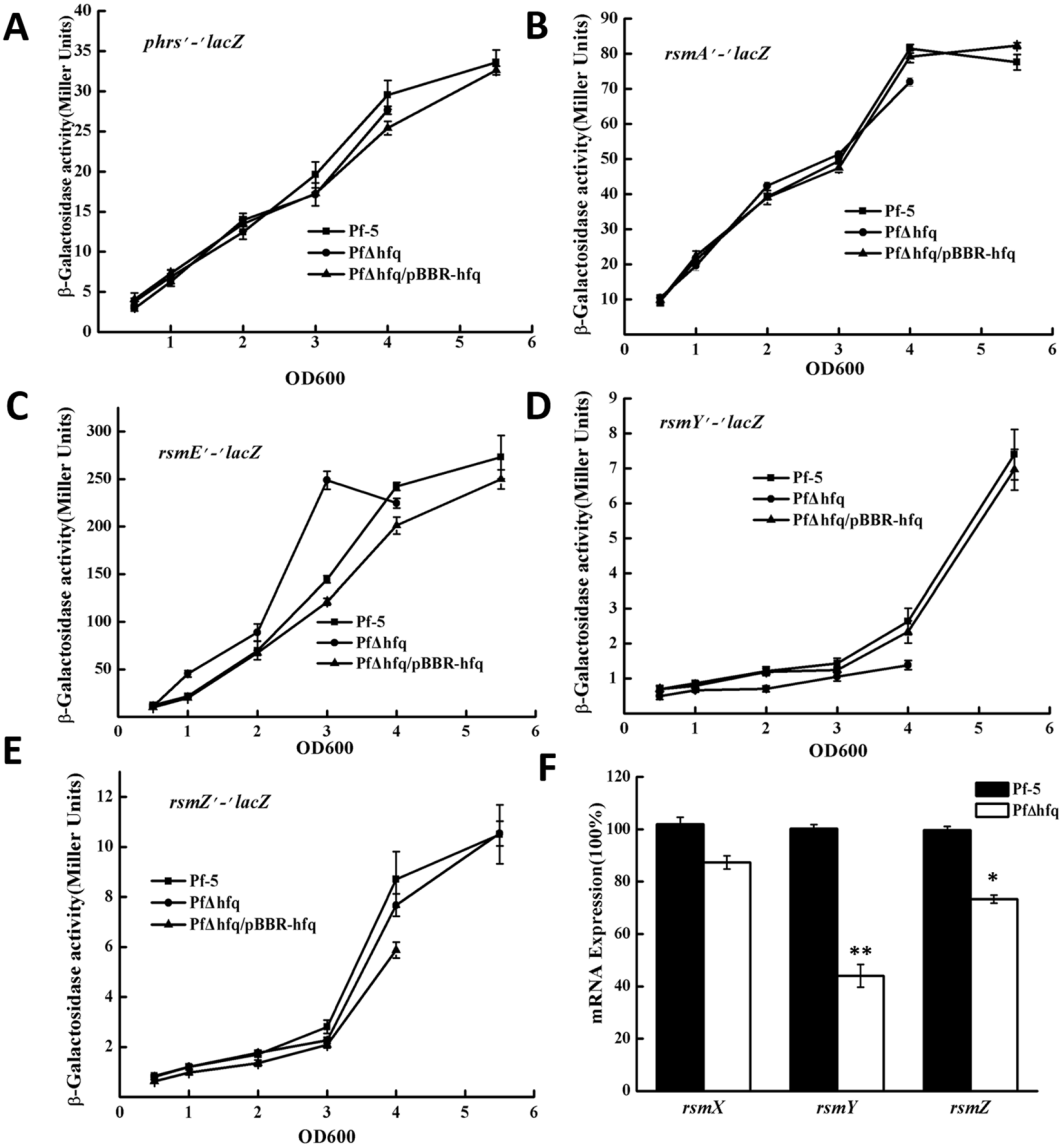
Effects of *hfq* on the activity of regulatory RNAs. (**A**) *phrS*, (**B**) *rsmA*, (**C**) *rsmE*, (**D**) *rsmY*, and (**E**) *rsmZ*. (**F**) qRT-PCR of relative expression of *rsmX, rsmY, and rsmZ* in *P. protegens* Pf-5 and the *hfq* mutant. The level of expression of *rsmX, rsmY, and rsmZ* mRNAs were measured after the bacterial growth reached stationary phase. Experiments were completed in triplicate. *P < 0.05, **P < 0.01 compared with the control group.

According to the literature, *hfq* can be directly associated with *phrs*, *rsmY*, and previous studies from our laboratory showed that in Pf-5, *rsmE* rather than *rsmA* can directly binds to *lipA* and affect the expression of *lipA*. Therefore, we determined the activity of *phrs*, *rsmA*, *rsmE*, *rsmY*, *rsmZ*, respectively, to see which genes were affected by Hfq (Fig. 3). The results show that the activity of *phrs*, *rsmA*, *rsmE* did not change after *hfq* knockout. However, the activity of *rsmZ* had mild changes, and the activity of *rsmY* changed robustly after *hfq* knockout. These results indicate that *hfq* may affect the expression of *lipA* via *rsmY* or *rsmZ*.

### Sequence interaction of hfq and rsmY

**Figure 4:**
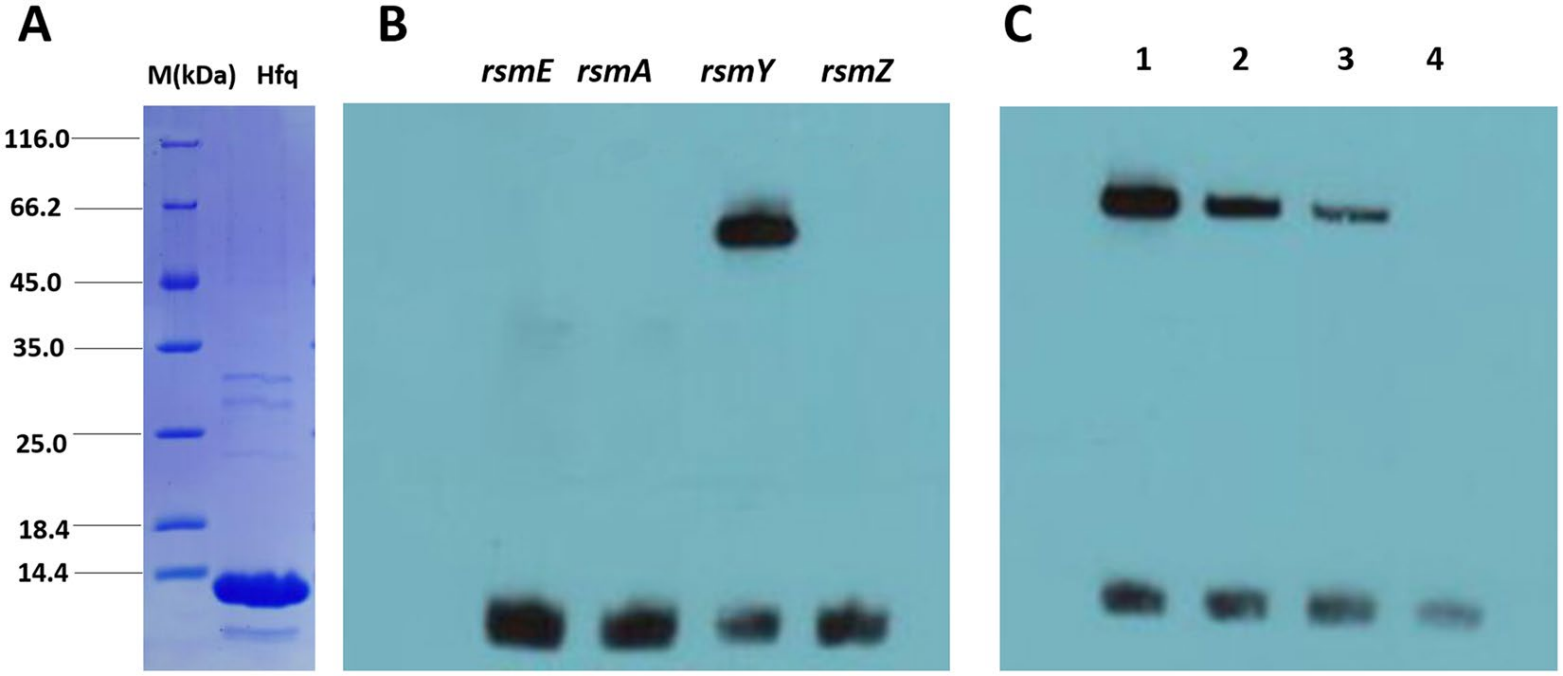
EMSA showing binding of *hfq* to the *rsmY* sequence. (**A**) Purified *Hfq* protein. (**B**) The 1-nM biotin-labeled DNA probe was incubated with purified Hfq protein in 20 μL binding buffer, and the Hfq-DNA complexes and free DNAs were cross-linked to the membrane by a 320-nm UV-light cross-linking instrument. Biotin-labeled bands were detected by chemiluminescent nucleic acid detection module. (**C**) Hfq protein directly bound to the *rsmY* sequence, but did not do so to *rsmA*, *rsmE*, and *rsmZ* sequences following increases in free *rsmY*. The different ratios of free *rsmY* to biotin-labeled *rsmY* used were 1:1, 50:1, 100:1, and 150:1 in groups 1 through 4.

To further investigate the relationship between *hfq* and *rsmY*, the *hfq* protein was purified in order to determine whether *Hfq* binds to *rsmY* or not, using EMSA. The EMSA results (Fig. 4) show that *hfq* did not bind to the promoter sequences of *rsmA, rsmE, rsmZ*, but did bid to rsmY (Fig. 4A and B). Furthermore, competitive EMSA (Fig. 4C **)** revealed that *hfq* concentrations and the amount of *rsmY* on the probe remained constant. Increasing the amount of free *rsmY* revealed direct association of *rfq* with *rsmY*. The different ratios of free *rsmY* to the *rsmY* bound to the probe were 1:1, 50:1, 100:1, and 150:1, in groups 1 through 4.

### Influence of Hfq on RsmY stability

**Figure 5:**
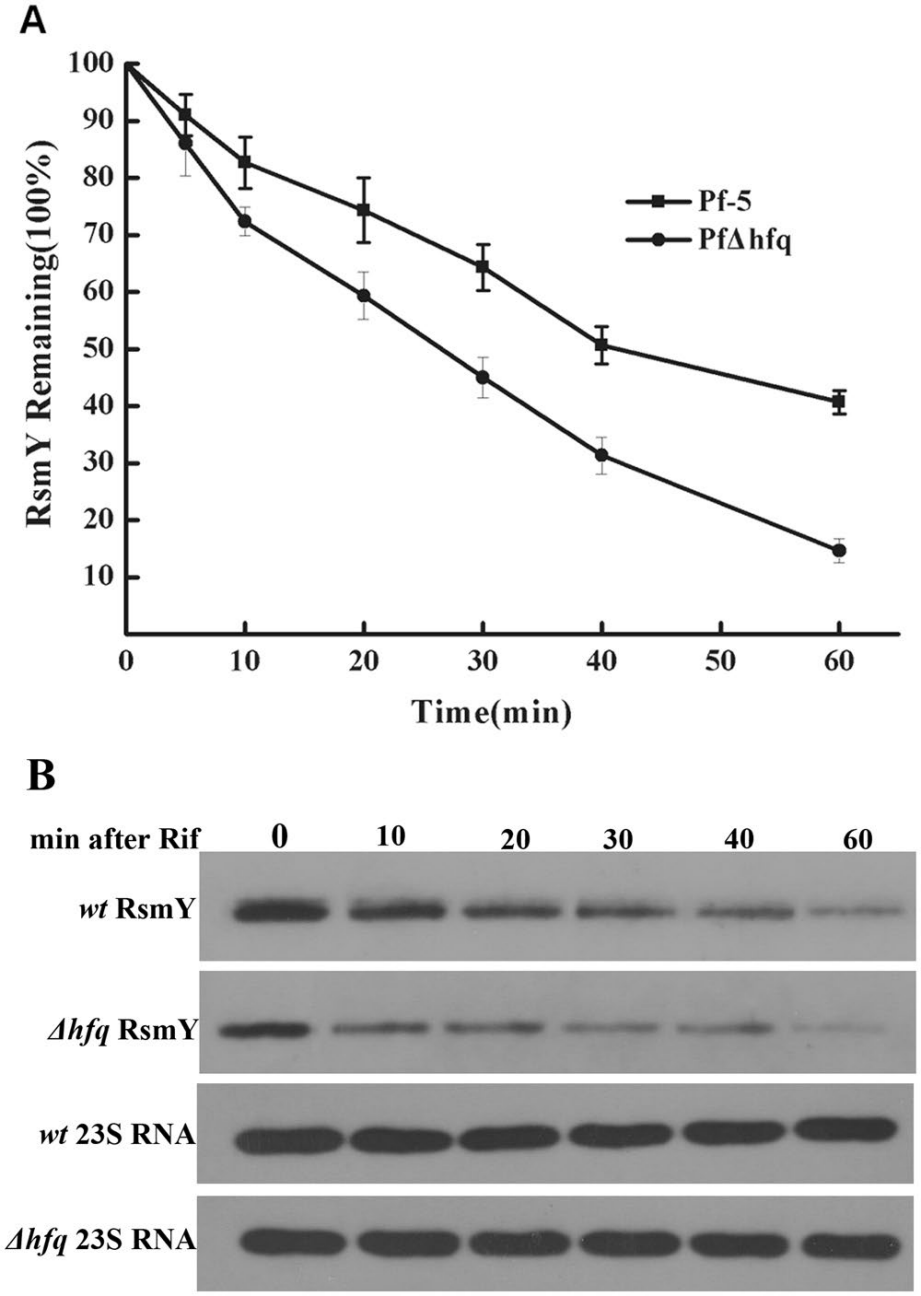
Effect of Hfq on the stability of RsmY. (**A**) Effects of rifampicin on RsmY stability in both wild-type *P. protegens* Pf-5 and the *hfq* mutant. (**B**) Representative Northern blot images following rifampicin treatment.

It is reported that the binding of Hfq and *rsmY* can maintain the stability of *rsmY*. However, in Pf-5, Hfq has not been studied. So we examined whether the stability of *rsmY* is different after rifampicin treatment. The results show that the stability of *rsmY* in *hfq* mutant was significantly lower than that of wild type after rifampicin treatment (Fig. 5). These results showed that Hfq affects the expression of *rsmY* by regulating the stability of *rsmY*, which in turn affects the expression of *lipA*.

### Influence of rsmY on lipA expression and lipase activity

**Figure 6:**
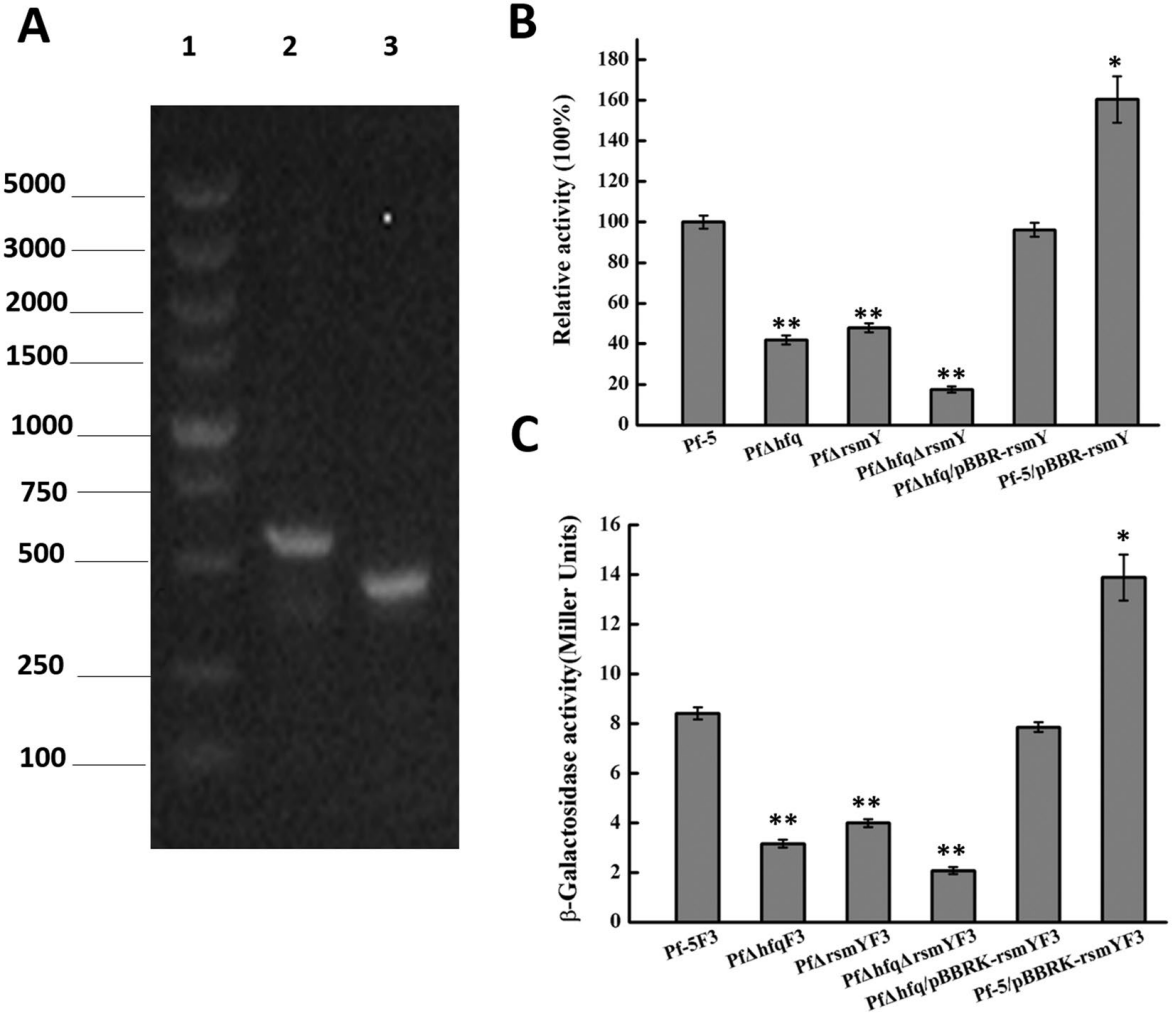
Effect of *rsmY* on *hfq* regulation of *lipA* expression. (**A**) Verification of *rsmY* knockout by PCR. Lane 1: DNA markers. Lane 2: wild type strain. Lane 3: mutant strains (PfΔrsmY). (**B**) Relative activity of whole-cell lipase activity following *hfq* and *rsmY* single or double knockout. Whole-cell lipase activity was measured following bacterial culture to the stationary phase. The relative lipase activity decreased to a greater extent following double knockout, than that observed in the single-gene knockout strain, indicating that *hfq* and *rsmY* both regulate lipase expression and activity, and that RsmY is capable of partially compensating for the effects of *hfq* knockout. (**C**) β-galactosidase activity following *hfq* and *rsmY* single or double knockout. β-galactosidase activity was determined following bacterial culture to the stationary phase. β-galactosidase activity was much lower in the double-knockout strain, and overexpression of *rsmY* in the *hfq* mutant enhanced β-galactosidase activity, indicating that the effects of *hfq* knockout on *lipA* expression was compensated for by *rsmY* expression. Experiments were completed in triplicate. *P < 0.05, **P < 0.01 compared with the control group.

After knocking out the *rsmY* gene (Fig. 6A), *lipA* expression was measured to verify the role of *rsmY* in regulating *lipA* expression. It was observed that *lipA* expression was reduced in cells where *hfq* was knocked out but the expression was considerably reduced in cells in which both *hfq* and *rsmY* knockout were effected (Fig. 6B and C). In addition, in the *hfq* mutant, re-incorporation of *rsmY* resulted in moderate recovery of *lipA* expression to near wild-type levels, and overexpression of *rsmY* resulted in *lipA* expression exceeding levels observed in wild-type strain and in the *hfq* mutant.

## Discussion

Many studies have shown that *hfq* is involved regulating the expression of multiple genes. It influences the activity of housekeeping genes containing Fe-S clusters, those controlling pyochelin and pyocin production. It also influences the activities of enzymes involved in degradation of aromatic compounds, alcohol dehydrogenase metabolism, and the ABC transport systems. Furthermore, *hfq* regulates QS-controlled genes, thereby regulating several virulence factors^10^. The expressions of global transcriptional regulators, like *Fur* ^11^, *RpoS* ^12^ and *H-NS* ^13^, are modulated by *hfq* in *E. coli*. However, the details regarding how these genes are regulated by *hfq* have remained unclear, and there are few reports regarding the regulation of *lipA* expression by *hfq*. The present study investigated the regulation *lipA* expression by *hfq* in *P. protegens* Pf-5 by knocking out the *hfq* gene. It was found that both *lipA* expression and lipase activity decreased significantly following *hfq* knockout. The *hfq* gene was also over-expressed in mutant PfΔhfq and wild-type *P. protegens* Pf-5. It was found that complementation of *hfq* restored *lipA* expression and lipase activity, and that *hfq* overexpression enhanced *lipA* expression and lipase activity in wild-type *P. protegens* Pf-5. Examination of the growth curve revealed that *hfq* knockout altered bacterial growth. These results evidently indicate that *hfq* regulates *lipA* expression and lipase activity in *P. protegens* Pf-5.

Electron microscope studies of *E. coli hfq* proteins^14–16^ and X-ray crystallography examination of the *hfq* protein from *P. aureus* ^14^ and *P. aeruginosa* ^17^, showed that 72 N-terminal amino acids are identical^18^. This finding led to the conclusion that the proteins belong to the *Sm*-like protein family which exhibit hexameric ring-shaped structures and recognize short U-rich stretches in primary RNA transcripts^19^. In eukaryotic cells, they are involved in RNA processing. In primary transcripts, a common *hfq*-binding motif exists, which is a stem-loop structure that precedes or follows an A/U-rich region^20–22^. Similar to *P. Protegens* CHAO, is GacS/GacA which regulates the expression of genes involved in secondary metabolism via *rsmX, rsmZ*, and *rsmY* in *P. protegens* Pf-5^5^. Transcriptions of *rsmZ* and *rsmY* are directly regulated by PAO1 and GacA to upregulate the expression of hundreds of genes in *P. aeruginosa* ^23^. In *P. aeruginosa*, PAO1, *hfq* and *rsmA* competitively associate with the GGA-repeat site of *rsmY* to regulate its expression^24^, and in *P. protegens* CHAO, the GGA-repeat site of *rsmY* is also a key *rsmA*-binding site^25^. Interestingly, the nucleotide sequence of *P. protegens* Pf-5 *rsmY* is exactly the same as that of *rsmY* from *P. protegens* CHAO. Therefore, it is reasonable to speculate that *hfq* regulates *rsmY* expression by associating with its GGA site. However, further studies will be necessary in order to accept or reject this hypothesis.

For *trans*-acting *hfq*-binding sRNAs in enteric bacteria, their regulatory function is associated with the RNA chaperone *hfq* at the post-transcriptional level. It is fairly well understood that the activity sRNA cannot be accomplished without the functional roles of *hfq*. The primary role of *hfq* is mostly to act as negative effector of translation by facilitating base-pairing between target mRNAs and sRNAs. However, it also acts as a positive effector in some cases^6, 26, 27^. The role it adopts, and the mechanism by which it accomplished that role depend on the specific bacterial system. For example, the majority of genes identified in microarray studies conducted following *hfq* knockout were not regulated by *hfq* in *P. aeruginosa* or in *E. coli* ^11^. Another role of hfq is to destabilize sRNA–mRNA hybrids on the one hand, through recruitment of RNase E near target mRNAs^28^. On the other hand, *hfq* stabilizes sRNAs by protecting them from ribonucleases^29, 30^. For example, in *P. aeruginosa*, sRNAs *PrrF1* and *PrrF2* play destabilizing roles on target mRNAs. Given that *hfq* can modulate *Fur* expression^11^ and that *Fur* controls *prrF1* and *prrF2* transcription, it follows that *hfq* affects the target genes of sRNAs indirectly^31^. The present study has shown that in *P. protegens* Pf-5, *hfq* not only regulates *rsmY* expression, but also destabilizes *rsmY*. However, the specific mechanism underlying this phenomenon remains unclear and warrants further investigations.

In a previous study, it was shown that *rsmE* rather than *rsmA* directly binds to the *lipA* promoter region to activate *lipA* transcription^5^. Therefore, further research is needed to investigate the interactions between the promoter region of *lipA* and *rsmX, rsmY, rsmZ, rsmA*, and *rsmE*, so as to elucidate the mechanisms underlying *lipA* expression via *rsmY*. The results obtained in the present study indicate that *lipA* expression in *P. protegens* Pf-5 is regulated at the transcriptional level by *hfq* through a mechanism involving direct binding of *hfq* to *rsmY*.

## Materials and Methods

### Bacteria, plasmids, and culture conditions

The bacteria and plasmids used in this study are listed in Table 1. *E. coli* was cultured by incubation at 37 °C. At the same time, *P. protegens* was cultured at 28 °C in lysogeny broth (LB; solid medium plus 1.5% agar). The antibiotics and concentrations used for *P. protegens* and *E. coli* culture were as follows: 40 μg/mL of kanamycin, 50 μg/mL of gentamicin, and 100 μg/mL of ampicillin. The concentration of sucrose used was 10% (w/v) when gene knockout was performed using the suicide plasmid pJQ200SK. Other components were isopropyl-β-D-thiogalactopyranoside (IPTG, 0.5 mM), ortho-nitrophenyl-β-D-galactopyranoside (4 mg/mL) and Taqaq (TaKaRa, Shiga, Japan). DNA ligase, plasmid preparation, restriction endonucleases, RNA reverse transcriptase, DNA gel extraction, and KOD Plus DNA polymerase (TaKaRa) were performed based on manufacturer’s protocol described in the commercial kits (Omega Bio-Tek, Doraville, GA, USA). Primers (synthetic oligonucleotides) were purchased from Anygene Biological Technology Co., Ltd. (Wuhan, China). Shanghai Sunny Biotechnology Co., Ltd. (Shanghai, China) provided DNA sequencing services. All molecular biology procedures were used based on standard methods.

**Table 1:** List of the bacteria and plasmids used in this study.

Strain/plasmid	plasmid Description	Reference or source
*E. coli*	Top10 *mcrA* (*mrr-hsdRMS-mcrBC*) 80*lacZ*M15 *lacX74 recA1 araD139*(a*raleu*)7*697galU galK rpsL*(Strr) *endA1 nupG*	Invitrogen
BL21(DE3)	F *ompT hsd S*B(rB mB) *dcm gal*(DE3)	Novagen
BL/pET-28a	BL21(DE3) with pET-28a; Km^r^	This study
BL/pET-hfq	BL21(DE3) with pET-hfq; Km^r^	This study
***P. protegens***		
Pf-5	Rhizosphere isolate; Ap^r^	32
Pf*Δhfq*	*hfq* derivative of Pf-5; Ap^r^	This study
Pf*ΔrsmY*	*rsmY* derivative of Pf-5; Ap^r^	This study
Pf*ΔhfqΔrsmY*	*Hfq and rsmY* derivative of Pf-5; Ap^r^	This study
Pf-5F3	pJQ003 conjugated into Pf-5; Gm^r^	This study
Pf-5F4	pJQ004 conjugated into Pf-5; Gm^r^	This study
Pf*Δhfq*F3	pJQ003 conjugated into Pf*Δhfq*; Gm^r^	This study
Pf*Δhfq*F4	pJQ004 conjugated into Pf*Δhfq*; Gm^r^	This study
Pf*ΔrsmY*F3	pJQ003 conjugated into Pf*ΔrsmY*; Gm^r^	This study
Pf*ΔrsmY*F4	pJQ004 conjugated into Pf*ΔrsmY*; Gm^r^	This study
Pf*ΔhfqΔrsmY*F3	pJQ003 conjugated into Pf*ΔhfqΔrsmY*; Gm^r^	This study
Pf*ΔhfqΔrsmY*F4	pJQ004 conjugated into Pf*ΔhfqΔrsmY*; Gm^r^	This study
***Plasmids***		
*Triparental mating*, pRK2073	Helper plasmid for triparental mating;Sp^r^	33
pJQ200SK	Suicide vector with *sacB* counterselectable marker used for homologous recombination; Gm^r^	34
pJQ*Δhfq*	pJQ200SK carrying a 1.9-kb *Xba*I/*Hind*III insert with a deletion in the coding region of *hfq*; Gm^r^	This study
pJQ*ΔrsmY*	pJQ200SK carrying a 1.7-kb *Xba*I/*Hind*III insert with a deletion in the coding region of *rsmY*; Gm^r^	This study
***Overexpression***		
pBBR1MCS-5	Broad-host-range vector; Gm^r^	35
pBBR1Km	*Nco*I*-BgI*II-digested kanamycin resistance cassettesubcloned in pBBR1MCS-5 digested with the same endonucleases	This study
pBBRKm	pBBR1Km with a 1,280-bp *Bam*HI*/Xba*I fragment harboring *lacI* ^q^-P_*lac*_; Km^r^	This study
pBBR-hfq	pBBR1MCS-5 with a 547-bp *Bam*HI/*Hind*III fragment harboring the codingregion of *hfq*; Km^r^	This study
pBBR-rsmX	pBBR1MCS-5 carrying a 183-bp *Bam*HI*/Hind*III fragment harboring the coding region of *rsmX*; Km^r^	This study
pBBR-rsmY	pBBR1MCS-5 carrying a 135-bp *Bam*HI*/Hind*III fragment harboring the coding region of *rsmY*; Km^r^	This study
pBBR-rsmZ	pBBR1MCS-5 carrying a 145-bp *Bam*HI*/Hind*III fragment harboring the coding region of *rsmZ*; Km^r^	This study
pBBRK-hfq	pBBRKm with a 547-bp *Bam*HI/*Hind*III fragment harboring the coding region of *hfq*; Km^r^	This study
pBBRK-rsmX	pBBRKm carrying a 183-bp *Bam*HI/*Hind*III fragment harboring the coding region of *rsmX*; Km^r^	This study
pBBRK-rsmY	pBBRKm carrying a 135-bp *Bam*HI/*Hind*III fragment harboring the coding region of *rsmY*; Km^r^	This study
pBBRK-rsmZ	pBBRKm carrying a 145-bp *Bam*HI/*Hind*III fragment harboring the coding region of *rsmZ*; Km^r^	This study
pET-28a	Expression vector carrying an N-terminal His tag thrombin-T7 tag configuration plus an optional C-terminal His tag sequence; Km^r^	Novagen
pET-hfq	pET-28a carrying a 305-bp *Nde*I*-Hind*III fragment harboring the coding region of *hfq*; Km^r^	This study
***Plasmid-borne lacZ fusion***		
pBBR01	pBBR1MCS-5 derivative with a translational *lipA*′-′*lacZ* fusion; Gm^r^	This study
pBBR02	pBBR1MCS-5 derivative with a transcriptional *lipA-lacZ* fusion; Gm^r^	This study
pBBR03	pBBR1MCS-5 derivative with a translational *rsmA*′-′*lacZ* fusion; Gm^r^	This study
pBBR04	pBBR1MCS-5 derivative with a translational *rsmE*′-′*lacZ* fusion; Gm^r^	This study
pBBR05	pBBR1MCS-5 derivative with a translational *rsmY*′-′*lacZ* fusion; Gm^r^	This study
pBBR06	pBBR1MCS-5 derivative with a translational *rsmZ*′-′*lacZ* fusion; Gm^r^	This study
pBBR07	pBBR1MCS-5 derivative with a translational *phrs*′-′*lacZ* fusion; Gm^r^	This study
***Chromosome-borne lacZ fusion***		
pJQ003	pJQ200SK derivative with a translational *lipA*′-′*lacZ* fusion; Gm^r^	This study
pJQ004	pJQ200SK derivative with a transcriptional *lipA-lacZ* fusion; Gm^r^	This study

### Gene knockout and complementation of hfq and rsmY in P. protegens Pf-5

The *rsmY* genes (900-bp and-800-bp, respectively) and the upstream and downstream fragments of the *hfq* (1000-bp and 900-bp, respectively) were fused by polymerase chain reaction (PCR) and digested with *Xba*I/*Hind*III along with the suicide plasmid pJQ200SK prior to their ligation to and construction of the vectors pJQΔhfq and pJQΔrsmY. The knockout vectors pJQΔhfq and pJQΔrsmY were then transferred into *P. protegens* Pf-5, and mutants were selected on 10% sucrose LB plates. The *P. protegens* Pf-5 harboring plasmid *pJQ200SK* was unable to grow on 10% sucrose plates, indicating that the double-recombination of the strains resulted in loss of plasmid pJQ200SK. Polymerase chain reaction (PCR) and sequencing confirmed the knockout of *hfq* and *rsmY* genes, and the strains were named PfΔhfq and PfΔrsmY, respectively. The *hfq* and *rsmY* double-gene knockout was achieved using the same methods. The knockout vector pJQΔrsmY was transferred into PfΔhfq, followed by selection for the *rsmY*-knockout mutant, and the double-gene knockout strain was named pfΔhfqΔrsmY. Here, pRK2073 was used as a helper plasmid, which was transferred into *P. protegens* Pf-5 using tri-parental hybridization.

The recombinant-expression plasmid pBBR-hfq was created to construct an *hfq* complementation strain following knockout. pBBR-*hfq* was constructed by ligating the promoter sequence and the 547-bp sequence containing the *hfq* gene into the shuttle plasmid *pBBR1MCS-5* of *Pseudomonas*–*E. coli* following *Bam*HI/*Hind*III digestion. Then pBBR-hfq was transferred into the PfΔhfq strain to generate the complementation strain pfΔhfq*/*pBBR-hfq. The plasmid pBBRK-hfq was constructed by ligating the 547-bp sequence containing the *hfq* gene and the promoter sequence into plasmid pBBRKm following *BamH*I*/Hind*III digestion. The same methods were then used to construct pBBR-rsmX, pBBR-rsmY, pBBR-rsmZ, pBBRK-rsmX, pBBRK-rsmY, and pBBRK-rsmZ.

### Construction of the promoter-lacZ reporter gene

The promoter-*lacZ* reporter gene was constructed for studying the regulation of lipase gene expression by *hfq*, by fusing the *lipA* promoter sequence with the *lacZ* sequence. PCR was used to amplify *lacZ* from the genomic DNA of *E. coli* BL21 (DE3), with the ‘*lacZ* amplicon (bp 22–3110 from the start site of translation) lacking the first seven codons and the sequence of Shine–Dalgarno (SD), whereas the amplicon of wild-type *lacZ* (bp 18–3110 from the start site of translation) contained the SD sequence. Into the plasmid pBBR1MCS-5, *lacZ* and ‘*lacZ* were inserted *Hind*III and *BamH*I cleavages, and cloned to generate the transcriptional-fusion plasmid pBBR02 and the translational-fusion plasmid PBBR01, respectively. The *lipA* gene was amplified using PCR, and following *Kpn*I and *Hind*III cleavages, the *lipA* amplicons (bp 613–18 from the start site of translation) were cloned into plasmid pBBR01 and cloned to generate plasmid pBBR03. Similarly, the inserted *lipA*′ amplicons (bp 613–12 from the start site of translation) in plasmid pBBR02 generated plasmid pBBR04 (Table 1). Into plasmid pJQ200SK, *lipA*′*-*‘*lacZ* and *lipA-lacZ* were inserted following pBBR03 and pBBR04 cleavages with *BamH*I *and Sph*I, and cloned to generate plasmids pJQ003 *and* pJQ004, respectively. The same methods were utilized to construct *rsmZ’-‘lacZ*, *rsmY’-‘lacZ*, *rsmE’-‘lacZ*, *rsmA’-‘lacZ*, and *phrs’-‘lacZ*.

### Reverse transcription (RT)-PCR analysis

*P. protegens* Pf-5 was cultured until the level of growth attained optical density value of about 5.5 at 600 nm (OD_600_) and thereafter, RNA was extracted using RNA extraction kit (CWBIO, Beijing, China). Following purification, 2 μg of the RNA was reverse-transcribed using random hexamer primers as described in Revert Aid kit instruction leaflet for first-strand cDNA synthesis (Thermo Fisher Scientific, Waltham, MA, USA). Real-time PCR machine (ABI 7500; Applied Biosystems, Foster City, CA, USA) was employed for quantitative RT-PCR (qRT-PCR) in 96-well plate with its default program (2 min at 50 °C and 10 min at 95 °C, followed by 40 cycles at 94 °C for 15 s and at 60 °C for 60 s). A total of 20 μL reaction mixture volume was used. The reaction mixture contained 6.4 μL of RNase-free water, 10 ng of final cDNA, 10 pM of each primer, and SYBR Green master mix (10 μL; Roche, Basel, Switzerland). There was a control with an aliquot of RNase-free water of 2.0 μL in each plate. Each plate contained three technical replicates. Prior to qRT-PCR evaluation of the *P. protegens* Pf-5 genes, PCR-efficiency curves as well as specific verification of the dissociated PCR-amplified candidate reference gene were determined. Using *rpoD* as an internal reference, differences in mRNA expression were determined.

### Expression and purification of Hfq protein

The 305-bp DNA fragment containing the entire *hfq* open reading frame sequence (261-bp) was amplified by PCR using *P. protegens* Pf-5 as a template. After cleavage with the restriction enzymes *Nde*I*/Hind*III, the generated fragment was inserted in the expression vector pET28a to produce the *hfq-*expression vector pET28a-hfq (Table 1). Into *E. coli* BL21 (DE3) cells, pET28a-hfq was transferred and the host cultured at 37 °C in LB containing 0.5 mM IPTG. Each *E. coli* BL21 (DE3) was allowed to grow and attain an OD_600_ of ~0.8. Then it was incubated for 20 h at 16 °C. The cells were thereafter pelleted by centrifugation and re-suspended in nickel A buffer [25 mM Tris–HCl (pH 8.0), 300 mM NaCl, and 20 mM imidazole] supplemented with 50 μM phenylmethyl sulfonyl, 1 μg/mL aprotinin, and 1 μg/mL leupeptin. After shaking the suspension for 30 min at 4 °C, an ultrasonic cell disruptor was used to lyse the cells. The lysate was allowed to percolate completely into a column of nickel-nitrilotriacetic acid agarose (GE Healthcare, Pittsburgh, PA, USA). The column was washed twice with 5 mL portions of nickel eluting buffer containing 500 mM imidazole, to elute the *hfq* protein. The purified *hfq* protein was then stored in a buffer containing 1 mM ethylenediaminetetraacetic acid (EDTA), 1 mM dithiothreitol (DTT), 200 mM NaCl and 20 mM Tris–HCl.

### RNA electrophoretic mobility shift assay (REMSA)

Light-shift chemiluminescent RNA EMSA kit (Thermo Fisher Scientific), REMSA was used for REMSA. RNA fragments of *rsmA*, *rsmE*, *rsmY*, *rsmZ* were synthesized *in vitro* and labeled with biotin. A 2-μL probe solution containing the respective biotin-labeled RNA fragment was mixed with 3 μL of purified *hfq* protein (1 mg/mL) in 10 μL of binding buffer [10 mM DTT, 10 mM MgCl_2_, 200 mM KCl, and 100 mM HEPES, pH 7.3], and placed for 10 min at room temperature to prevent non-specific binding of the protein and probe. In studies on competitive binding, unlabeled probe concentrations were 50-fold, 100-fold, and 150-fold higher than that of the labeled-probe. Binding buffer (1 μL; colorless; 10 × ) was added and mixed immediately. Pre-electrophoresis was conducted for 30 min with 0.5 × TBE (Tris/borate/EDTA) as the electrophoretic solution at 80 V. The electrophoretically-separated protein-RNA conjugates were bound to a positively-charged nylon membrane (Ambion; Thermo Fisher Scientific). The membrane was cross-linked by the free RNAs and transferred *hfq*-RNA complexes when exposed to UV light at 320 nm. The biotin-labeled nucleic acid bands on the membrane were detected by chemiluminescence (Thermo Fisher Scientific). While on nylon membrane, the transferred biotin-labeled RNAs were visualized by using the activated conjugate of stabilized streptavidin and horseradish peroxidase (HRP). In order to produce light of high sensitivity, the HRP was allowed to act on luminol-based substrate. The luminescent membrane was exposed to X-ray film after remaining in a film cassette for 20–30 seconds.

### Determination of rsmY abundance and stability

The strains *P. protegens* Pf-5 and PfΔhfq were used to determine the stability and steady-state level of *rsmY*. It was added at an OD_600_ value of 4.0 to 500 µg/mL of rifampicin (final concentration). Rifampicin was also added to the total RNA isolated from 4 ml aliquot at 0, 10, 20, 30, 40, and 60 min. Aliquots (4-mL portions) were withdrawn. With 2 µg of total RNA, primer extension technique was used to determine *rsmY* concentrations with AMV reverse transcriptase (Promega, Durham, NC, USA).

### Northern blot

Denaturing gel composed of urea and polyacrylamide (8.3 M urea, 8% acrylamide, and 0.2% bisacrylamide) was used for electrophoretic separation of RNA and subjected to northern blot in 1 × TBE buffer [50 mM Tris-borate (pH 8.3) and 1 mM EDTA]. The molecular-weight markers (low-range RNA ladder; Fermentas, Waltham, MA, USA) corresponding to the band in a lane was excised and stained with 5 mg/mL of ethidium bromide. It was then photographed under UV light beside a reference ruler. The remaining gel was electroblotted for 20 min in 1 × TBE buffer onto a Hybond-N membrane at 150 mA (15–25 V). Nucleic acids in the membranes were cross-linked by exposure to UV light for 5 min. Then 2 × SSC (1 × SSC contains 0.15 M NaCl and 15 mM sodium citrate) was used to wash all membranes (Sambrook and Russell, 2001). Northern hybridizations were performed according to recommended protocols (DIG filter hybridization; Roche) for using digoxigenin (DIG)-labeled DNA probes.

### β-Galactosidase assay

The β-galactosidase activity assay was performed as previously described^36^. The enzyme activity was normalized in Miller units of bacterial culture to the OD_600_ value. In order to induce the expression of strains containing pBBR1MCS-5 or pET-28a derivatives, 0.1 mM IPTG was added to cultures.

### Lipase-activity assay

In view of the fact that LipA is an intracellular lipase, LipA activity was measured as the activity of whole-cell lipase. According to previously described methods [5], bacterial samples were prepared and 30 μL of *p*-nitrophenyl caprylate [pNPC; 2.9 mL 50 mM Tris–HCl (pH 9.0) and pNPC (10 mM pNPC in acetonitrile)] was used to determine lipase activity. The reaction mixture containing 70 μL of the cell sample was pre-heated for 5 min at 55 °C and centrifuged at 12,000 rpm for 2 min at 4 °C. The amount of pNP released in the supernatant was determined spectrophotometrically by measuring absorbance at 600 nm. One unit of enzyme activity (U) was defined as the amount required to release 1 μmol of *p*-nitrophenol/min. Lipase activity was expressed as U/mL*OD_600_.

### Ethical approval

This article does not contain any studies with human participants or animals performed by any of the authors.
